# Zinc Ion-Dependent Peptide Nucleic Acid-Based Artificial Enzyme that Cleaves RNA—Bulge Size and Sequence Dependence

**DOI:** 10.3390/molecules22111856

**Published:** 2017-10-29

**Authors:** Merita Murtola, Alice Ghidini, Pasi Virta, Roger Strömberg

**Affiliations:** 1 Department of Chemistry, University of Turku, 20014 Turku, Finland; pamavi@utu.fi; 2 Department of Biosciences and Nutrition, Karolinska Institutet, Novum, 141 83 Huddinge, Stockholm, Sweden; alice.ghidini@pharma.ethz.ch

**Keywords:** artificial ribonuclease, oligonucleotide, PNA, PNAzyme, phosphodiester cleavage, sequence selectivity, RNA bulge

## Abstract

In this report, we investigate the efficiency and selectivity of a Zn^2+^-dependent peptide nucleic acid-based artificial ribonuclease (PNAzyme) that cleaves RNA target sequences. The target RNAs are varied to form different sizes (3 and 4 nucleotides, nt) and sequences in the bulge formed upon binding to the PNAzyme. PNAzyme-promoted cleavage of the target RNAs was observed and variation of the substrate showed a clear dependence on the sequence and size of the bulge. For targets that form 4-nt bulges, we identified systems with an improved efficacy (an estimated half-life of ca 7–8 h as compared to 11–12 h for sequences studied earlier) as well as systems with an improved site selectivity (up to over 70% cleavage at a single site as compared to 50–60% with previous targets sequences). For targets forming 3-nt bulges, the enhancement compared to previous systems was even more pronounced. Compared to a starting point of targets forming 3-nt AAA bulges (half-lives of ca 21–24 h), we could identify target sequences that were cleaved with half-lives three times lower (ca 7–8 h), i.e., at rates similar to those found for the fastest 4-nt bulge system. In addition, with the 3-nt bulge RNA target site selectivity was improved even further to reach well over 80% cleavage at a specific site.

## 1. Introduction

Therapeutic oligonucleotides (ONs) provide an opportunity for treating serious, life-threatening diseases with limited options using traditional low molecular weight molecules and antibody drugs [1]. Although there are many ongoing clinical trials and some ON drugs [2], ON therapeutics has not quite lived up to the early expectations, especially regarding the time frame for development. There has been a substantial number of promising results [1,2] but it is recognized that the most severe limitation in ON therapeutics is their delivery (or rather lack thereof) to the chosen site of action [3,4]. 

Moreover, antisense oligonucleotide (AON) action based on a 1:1 binding stoichiometry may require a relatively high dosage of ON to silence the target mRNA transcript effectively [5]. Efficacy of therapeutic ONs is more readily achieved if catalytic cleavage of the target RNA is obtained, which can occur if native enzymes (e.g., RNAse H for antisense and RNA-induced silencing complex (RISC) for siRNA) can be recruited. Chemically modified antisense oligonucleotides often do not retain the ability to activate native enzymes. It is also not completely clear if in some circumstances the activity and/or concentration of the host enzymes can become limiting.

ON-based artificial nucleases (OBANs) [6,7,8,9,10,11] have the potential to affect RNA levels and thereby also diseases, while at the same time being independent of native enzymes with the freedom to use many available ON modifications. RNA sequence and secondary structures affect the reactivity of the phosphodiester bonds and studies have been done to evaluate structural parameters to support the development of sequence-specific RNA cleaving agents that mimic natural restriction enzymes [12,13]. RNA oligonucleotides are considerably more reactive in the single stranded form (also as bulges or loops) than as a duplex, and the cleavage of the phosphodiester bonds has a strong dependence on the sequence [14,15,16]. Furthermore, the molecular environment within the RNA bulge, including the size, bulge sequence, stacking of the nucleobases, and orientation of the phosphodiester bonds within, has a considerable influence on the overall reactivity of the phosphodiester bonds. It is also preferable to cleave the target RNA in a central part of the RNA sequence so that RNA fragments can readily dissociate [17,18,19].

We have designed artificial nucleases based on the concept that the modified oligonucleotide part of the artificial nuclease recognizes the target RNA upon hybridization and forces the RNA to form a bulge. This concept enabled the development of artificial enzymes capable of cleaving target RNA sequences with turnover [20,21,22]. Previous efforts have also resulted in the development of peptide nucleic acid-based artificial nucleases (PNAzymes) [23,24,25,26]. PNAzymes using Cu^2+^ as a co-factor were shown to act as RNA restriction enzymes with high site selectivity and half-lives of cleavage down to 15 to 30 min, at a 1:1 ratio of PNAzyme to RNA [27,28]. It was also found that the rate of cleavage with the Cu^2+^ PNAzymes was highly dependent on the sequence of the non-paired bulge sequence that were formed in the target RNA [27]. PNAzymes with Zn^2+^ as a co-factor was shown to cleave target RNA within 3- and 4-nt bulges with a half-life as low as 11 h [26]. However, these zinc-based systems were not studied with respect to how the bulge sequence may affect the rate and site selectivity of the RNA cleavage. In the present study, we report on how the size and sequence of formed 3- and 4-nt bulges affect the rate and site selectivity of RNA cleavage by a Zn^2+^-dependent PNAzyme. This study was motivated by the fact that we found the bulge sequence to have a substantial effect on the rate of cleavage in Cu^2+^-based systems [27]. In our current development towards Zn^2+^-based artificial nucleases that are more biocompatible than copper-based systems, it is essential to investigate different RNA sequences in order to determine how this can effect PNAzyme action. 

## 2. Results and Discussion

**Figure 1:**
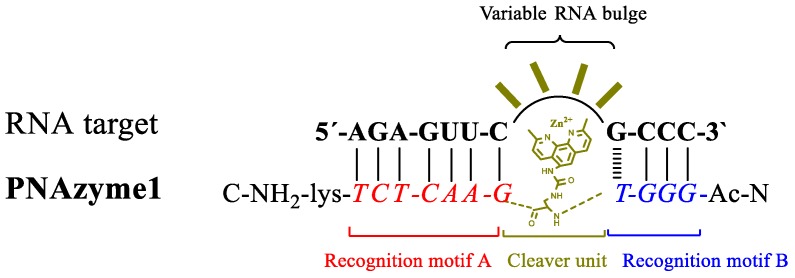
Schematic representation of the concept of bulge forming RNA/peptide nucleic acid-based artificial ribonuclease (PNAzyme) complex.

Metal ions, the stacking interactions within an RNA/PNA duplex, and the formed RNA bulge may have a structural role affecting the reactivity of phosphodiester bonds within the bulge, making the activity of PNAzyme complexes quite sensitive to the sequence of the RNA molecule and also dependent on the metal co-factor used. For the evaluation of structural factors in the bulge, we chose to utilize a PNAzyme [26] that has been shown to cleave a model of the Leukemia-related bcr/abl mRNA in the presence of zinc ions. This PNAzyme cleaves the target RNA in a catalytic fashion with a half-life of down to 11 h when an AAAA bulge is formed [26]. Scission of the bcr/abl mRNA takes place in several positions within the bulged out RNA. As a starting point for this study, we selected systems where the RNA/**PNAzyme1** complex will form 4-nt bulges in the RNA part (Figure 1). 

*Four-bulge forming target RNAs*: We performed a relatively systematic variation of the initial 4-adenosine bulge forming RNA substrate (**RNA1**) and investigated how the bulge sequence influenced the rate and selectivity of the RNA cleavage reaction conducted by the Zn^2+^-dependent **PNAzyme1**. Thus, 21 additional RNA sequences (**RNA2**–**22**, Table 1) were separately treated with **PNAzyme1**. The reactions were performed using equimolar amounts of **PNAzyme1** and RNA (both at 4 μM) in the presence of 100 μM Zn^2+^ (pH 7.4 and 37 °C) following conditions described in previous studies [26]. Aliquots were withdrawn from the reaction mixtures at 3 and 24 h. Variation of the 4-adenosine bulge sequence by replacing one adenine base with one of the other three natural nucleobases (**RNA2**–**4**, **RNA5**–**7**, **RNA11**–**13**, and **RNA14**–**16**) revealed a clear dependence of the cleavage rate on the bulge composition (Table 1). Further variation of the bulge sequence (**RNA17**–**22**) confirmed the substantial influence of the bulge sequence on the rate of cleavage. 

**Figure 2:**
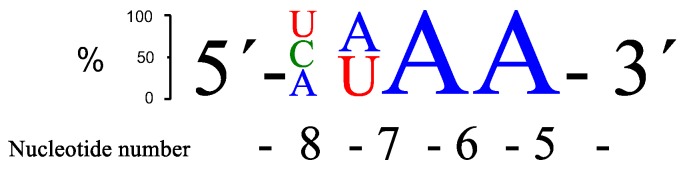
A schematic presentation of the three overall most prominent kinetic preferences for 4-nt bulge RNA targets (AUAA, CAAA and UUAA) upon **PNAzyme1**-mediated cleavage.

Replacement of the adenosine at position 5, adjacent to the GT wobble base pair (**RNA2**–**4**), decreased the rate of cleavage regardless of the base substituted. The main cleavage site remained the same and was located between positions 5 and 6, i.e., the nucleotide at position 6 provided the 2′-hydroxyl nucleophile for the phosphodiester cleavage reaction and resulted in the formation of a 2′, 3′-cyclic phosphodiester and release of the 3′-end fragment with a free 5′-hydroxyl. Replacing the adenosine at position 6 (**RNA5**) with guanosine was somewhat tolerated, while the cleavage rate dropped substantially if the purine bases were replaced by a pyrimidine in this position (**RNA6**–**7**). The change to a guanosine at position 5 was also better tolerated than the change to a pyrimidine base (**RNA2**–**4**). These effects were confirmed by the data obtained with **RNA8**–**10** and suggest that either stacking between base 5 and 6 and/or similar interaction with the neocuproine affected the rate of cleavage, and that stacking to the G in the wobble base pair may be important. This is in clear contrast to the results obtained for position 7, where a base change to a pyrimidine (**RNA12**–**13**) in the target RNA led to an even higher rate of cleavage (change to G (**RNA11**) in this position did not affect cleavage efficiency much). The cleavage rate is less sensitive to variation at position 8 in the bulge, although G and A seem to be less favoured. **RNA13** (AUAA), **RNA15** (CAAA), **RNA17** (UUAA), and **RNA20** (AUGA) gave the most cleavage after 24 h, closely followed by a few more sequences. The sequence preferences with respect to rate of cleavage are summarized in Figure 2. 

**Figure 3:**
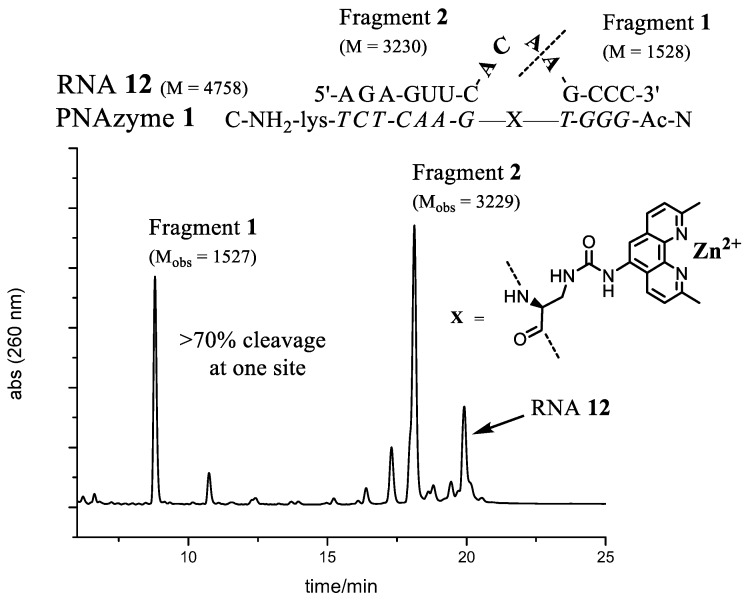
The complex between **PNAzyme1** and substrate **RNA12**, as well as the Ion-Exchange High Performance Liquid Chromatography (IE-HPLC) chromatogram displaying 72% relative cleavage selectivity of the target RNA (24 h incubation time, 1:1 PNAzyme1/RNA12, 4 μM of each, 100 μM Zn^2+^, pH 7.4, 37 °C). The cleavage site is indicated by the dashed line. M designates calculated molecular weight and M_obs_ denotes the molecular weights of isolated fragments as measured by Electrospray Ionisation Time-of-Flight mass spectrometry (ESI-TOF). Fragment 1 contains a free 5′-hydroxyl group and Fragment 2 contains a 2′, 3′-cyclic phosphate.

The best site selectivity in the 4-nt bulge systems was achieved with the bulge sequence -ACAA- (**RNA12**), showing more than 70% selectivity for cleavage at one site (Figure 3), which was an improvement compared to the AAAA bulge system [26]. The ACAA bulge was also cleaved at a higher rate, though not as efficiently as, e.g., the AUAA bulge. Several similarities in the preferences for bulge sequences can be seen when comparing the rate of cleavage with Cu^2+^-based PNAzymes [27]. Although differences were larger for the latter, the zinc- and copper-based PNAzymes seem to show a similar nucleobase-related rate dependence at positions 5–7 of the RNA bulge and AUAA was also the most favoured sequence for the Cu^2+^-based cleavage. In contrast, position 8 suggested a preference for A in copper-based systems, while the zinc-based systems equally accept pyrimidine bases.

*Three-bulge forming target RNAs*: OBANs can display different preferences for different RNA bulge sizes, and the sequence dependence has not been studied for 3-nt bulges. To explore this further, we investigated the influence of the 3-nt bulge sequence on RNA target cleavage by the Zn^2+^-based **PNAzyme1**. The starting point was again an all-adenosine bulge where each one of the adenines were systematically replaced by other nucleobases. This revealed an even higher dependence of the rate of cleavage on the bulge composition than that observed for the 4-nt bulges (Table 2).

The 3A bulge RNA (**RNA23**, Table 2) was cleaved at a lower rate than the corresponding 4A bulge target (**RNA1**, Table 1). The results of this experiment, taken on their own, could indicate that the smaller size of the RNA bulge affects the cleavage efficiency negatively. However, as further experiments with other sequences were continued, this became less evident. For the nucleotide adjacent to the wobble base pair (position 5) adenine is clearly the preferred base (cf **RNA23** and **RNA27**, **RNA24** and **RNA30**, **RNA26** and **RNA37**). For the central position (position 6) the presence of a uracil base in the RNA target appears to give a higher rate of cleavage (**RNA24**). This is the position that provides the 2′-hydroxyl nucleophile at the main cleavage reaction site, as evidenced by the main fragments formed. Changing the adenine to guanine (**RNA25**) gives a smaller enhancement, while change to a cytosine (**RNA26**) led to a slightly decreased rate.

**Figure 4:**
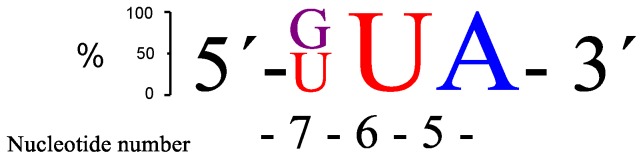
A schematic presentation of the two overall most prominent kinetic preferences for 3-nt bulge RNA targets (GUA and UUA) upon **PNAzyme1**-mediated cleavage.

Replacing the adenine in position 7 (**RNA28**) led to a notable increase in the rate of cleavage. Out of the **RNA23**–**28** target RNAs, the AUA bulge (**RNA24**) gave the best site selectivity with more than 80% of the cleavage taking place at the phosphodiester connecting A-5 and U-6. Further investigations were continued by systematically changing the bases within the AUA bulge region of **RNA24**. Changing the base at position 5 neighbouring the GT wobble base pair generated a decrease in the cleavage activity (**RNA29**–**31**). This correlated with the 4-nt bulge systems where adenine was the preferred choice in the corresponding position (Figure 2). Replacing adenine in position 7 with any of the other three standard bases pushed the rate of cleavage upwards. These 3-nt bulge PNAzyme complexes (**RNA32**–**34**) displayed rates comparable to the fastest 4-nt bulge (AUAA, **RNA13**) system. Three additional RNAs (**RNA35**–**37**) were also evaluated, but cleavage activity decreased. The two RNA targets that seemed to be cleaved most efficiently were those with either GUA (**RNA32**) or UUA (**RNA34**) sequences in the bulge, as is schematically shown in Figure 4. 

**Figure 5:**
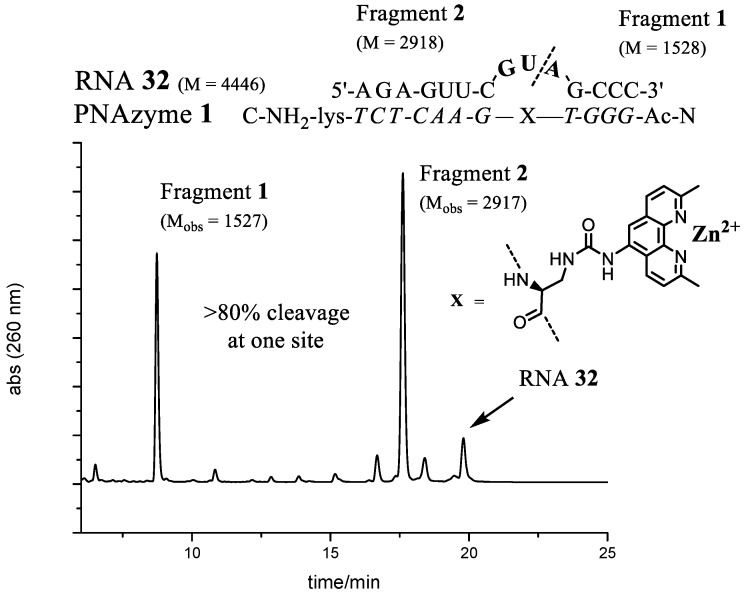
The complex between **PNAzyme1** and substrate **RNA32**, as well as the IE-HPLC chromatogram displaying 82% relative cleavage selectivity of the target RNA (24 h incubation time, 1:1 PNAzyme1/RNA32, 4 μM of each, 100 μM Zn^2+^, pH 7.4, 37 °C). The cleavage site is indicated by the dashed line. M designates calculated molecular weight and M_obs_ denotes the molecular weights of isolated fragments as measured by mass spectrometry (ESI-TOF). Fragment 1 contains a free 5′-hydroxyl group and Fragment 2 contains a 2′,3′-cyclic phosphate.

There is some, but not complete, correlation between the cleavage rate and site selectivity of **PNAzyme1**-mediated cleavage reactions. The highest site selectivity of 3-nt bulge systems studied was achieved with the bulge sequences AUA (**RNA24**), GUA (**RNA32**), and UUA (**RNA34**), which resulted in over 80% relative site selectivity (e.g., GUA in Figure 5). This is a significant improvement compared to other reported 3-nt [26] systems and to the 4-nt Zn^2+^-based systems in Table 1.

## 3. Materials and Methods 

Peptide nucleic acid monomers, Fmoc-PNA-A(Bhoc)-OH, Fmoc-PNA-G(Bhoc)-OH, Fmoc-PNA-C(Bhoc)-OH and Fmoc-PNA-T-OH, were purchased from Link Technologies Ltd. (Glasgow, UK). Rink Amide resin (ChemMatrix, 0.47 mmol/g) was purchased from Biotage (Uppsala, Sweden). Solvents and reagents for solid-phase synthesis; Iris Biotech GmbH (Marktredwitz, Germany) (2,3-diaminopropionic acid (Fmoc-L-Dap(Mtt)-OH), Fmoc-L-Lys(Boc)-OH, trifluoroacetic acid (TFA), triisopropylsilane (TIS), dichloromethane (DCM) and *N*-methylpyrrolidone (NMP)), Acros Organics (NJ, US) (diethyl ether), Sigma-Aldrich (St. Louis, MO, USA) (piperidine, phenyl chloroformate, 4-methylmorpholine), Merck-Millipore (Burlington, MA, USA) (ethyl cyano(hydroxyimino) acetate (Oxyma) and *N*,*N*’-diisopropylcarbodiimide (DIC)), Alfa Aesar (Haverhill, MA, USA) (2,6-lutidine), VWR (Radnor, PA, USA) (acetonitrile, MeCN), and Applied Biosystems (Foster City, CA, USA) (acetic anhydride). 5-amino-2,9-dimethyl 1,10 phenanthroline was synthesized as previously reported [30]. All chemicals used in the RNA cleavage assays were of molecular biology grade. All RNA oligomers were purchased from Dharmacon (Lafayette, CO, USA). RNA oligomers 5′-AGAGUUCAAAGCCC-3′ (**RNA23**) and 5′-AGAGUUCAUAGCCC-3′ (**RNA24**) were bought as IE-HPLC-purified and desalted. All other RNAs were deprotected according to manufacturer′s protocol (2’-ACE protecting groups), purified by the Ion-Exchange High Performance Liquid Chromatography (IE-HPLC) and desalted by Reversed Phase HPLC (RP-HPLC). IE-HPLC purifications were performed using an analytical Thermo Scientific DNAPac PA-100 BioLC (4 × 250 mm) column (Waltham, MA, USA). A linear gradient of 0–35% buffer B over 15 min was used with a flow rate of 1.5 mL/min at 60 °C, (Buffer A) 20 mM NaOAc in 30% MeCN/aq., (Buffer B) 20 mM NaOAc, 0.4 M LiClO_4_ in 30% MeCN/aq. UV detection was carried out at 260 nm. Purified RNAs were desalted by reverse-phase HPLC on a Jasco HPLC system using the Supelco Discovery BIO Wide Pore C18-5.5 μm (250 × 4.6 mm) column (Sigma-Aldrich). A linear gradient of 0–37% buffer B over 20 min was used with a flow rate of 1 mL/min at 50 °C, (Buffer A) 50 mM triethylammonium acetate (TEAA) in water (pH 6.5) and (Buffer B) 50 mM TEAA in water (pH 6.5)-acetonitrile (1:1, *v*/*v*). Purified and desalted RNAs were lyophilized three times before use and stored as frozen solution. Concentrations of RNA sequences were determined by UV absorption at 260 nm on a Varian Cary 300 UV-vis dual beam spectrophotometer (Varian) and calculated from extinction coefficients obtained by the nearest neighbour approximation [31].

### 3.1. Synthesis of Peptide Nucleic Acid-Based Artificial Ribonuclease (**PNAzyme1**)

Chimeric PNA oligomer was prepared on a Biotage Initiator+ Alstra microwave peptide synthesizer. The sequence was assembled automatically on Rink Amide ChemMatrix resin (0.47 mmol/g) on a 10-μmol scale in a 5-mL reactor vial using 9-fluorenylmethoxycarbonyl (Fmoc) chemistry under inert gas (N_2_). Fmoc deprotection was performed at room temperature (RT) in two stages by treating the resin with piperidine-NMP (1:4, *v*/*v*) for 3 min followed by piperidine-NMP (1:4, *v*/*v*) for 10 min. The resin was then washed with NMP (×4). PNA couplings were performed using 4 eq. of PNA monomer (or Lys or Dap monomers), 4 eq. Oxyma, and 4 eq. DIC in NMP. A coupling time of 6 min at 75 °C (microwave) was employed and the resin was washed with NMP (×4). This was followed by a capping step using NMP-lutidine-acetic anhydride (89:6:5, *v*/*v*/*v*) for 2 min and then washing with NMP (×4). After the synthesis cycles were completed, the resin was washed with NMP (×5) and DCM (×6), and then dried. Prior to post-conjugations, the methyltrityl (Mtt) protection group of 2,3-diaminopropionic acid was removed by subjecting the solid-supported PNA to 1% trifluoroacetic acid (TFA) in dichloromethane (DCM) for 5 × 1 min, followed by washing with DCM and NMP. 5-amino-2,9-dimethyl-1,10-phenanthroline (10 mg, 45 μmol) was dissolved in DCM (200 μL). 4-methylmorpholine (NMM, 10 μL, 90 μmol) and phenyl chloroformate (5 μL, 40 μmol) was added to the solution, and the reaction mixture was agitated for 1 h. The mixture was partitioned between DCM and H_2_O (200 μL). The organic phase was added to the solid support bound PNA with 50 μL NMM and reaction was incubated overnight at room temperature. The support was filtered and washed with NMP and DCM. PNA was cleaved from the solid support using a cocktail of TFA-H_2_O-TIS (95:2.5:2.5, *v*/*v*/*v*/*v*) for 1.5 h at room temperature. The PNA product was evaporated to dryness by a nitrogen flow, diluted with deionized (MilliQ) water and evaporated to dryness under reduced pressure. The crude product was partitioned between diethyl ether and water (×3), and the water phase was evaporated to dryness.

RP-HPLC purification and analysis was carried out on a Ascentis Express Supelco Peptide ES-C18 column (2.7 μm, 150 × 4.6 mm) with a linear gradient elution of 27% B over 30 min at 60 °C, using a flow rate of 1.0 mL/min and UV detection at 260 nm. The following solvent system was used: solvent A, water containing 0.1% TFA; solvent B, 50% MeCN: water containing 0.1% TFA (1:1; *v*/*v*). Collected products were evaporated to dryness and lyophilized from water (×3). Mass spectrometry of the collected products was performed on a ESI-TOF mass spectrometer in positive ion mode using a solution of acetonitrile-water (1:1, *v*/*v*) containing 0.1% formic acid. ES-MS *m*/*z*: mass calculated for **PNAzyme1** C_145_H_180_N_72_O_38_ [M]^+4^ calcd 3539, found 3539. 

### 3.2. RNA Cleavage Assay Using Anion Exchange (IE) HPLC

RNA cleavage reactions were carried out in sealed tubes immersed in a thermostated water bath (37 °C). These experiments were performed in 10 mM HEPES buffer at pH 7.4, containing 0.1 M NaCl and 100 μM Zn^2+^ (110 μM Zn^2+^ , 10 μM ethylenediaminetetraacetic acid (EDTA), resulting in 100 μM accessible zinc ions). **PNAzyme1** is known to cleave phosphodiester bonds efficiently in the presence of copper ions [27]. EDTA was used to eliminate possible copper contamination. All reactions were performed in equimolar concentrations (4 μM) of substrate RNA and PNAzyme. The samples were prepared at room temperature by adding the appropriate amounts of deionized (MilliQ) water, buffer concentrate, substrate RNA, metal ion stock solution, and PNAzyme to achieve the final concentrations. After the addition of all components, at 3-h and 24-h time intervals, 70-μL aliquots were withdrawn from the reactions and quenched by adding 30 μL of a solution of 1 mM EDTA in 30% aq. MeCN. The samples were analysed by anion exchange HPLC (IE-HPLC). IE-HPLC analysis was carried out on a Dionex NucleoPac PA-100 (4 × 250 mm) column with a linear gradient elution of 0–45% buffer B over 30 min at 60 °C. A flow rate of 1.5 mL/min was used and UV detection was carried out at 260 nm. The following solvent system was used: (A) 20 mM NaOAc in 30% aq. MeCN and (B) 20 mM NaOAc, 0.4 M LiClO_4_ in 30% aq. MeCN. Cleavage of RNA substrates were obtained by the quantification of the remaining RNA and the sum of all the formed fragments detected in the IE-HPLC analysis. Further examples of chromatograms can be found in Supplementary materials (Figures S1 and S2).

### 3.3. Determination of RNA Cleavage Sites

RNA cleavage reactions were performed as above. The main RNA fragments were collected from IE-HPLC runs and desalted as described earlier. Mass spectrometry of the collected fractions was performed in negative ion mode using a solution of MeCN–water (1:1, *v*/*v*)**. **ES-MS *m*/*z*: **RNA12** (-ACAA-) Fragment1 C_47_H_61_N_19_O_32_P_4_ [M]^−2^ calcd 1528, found 1527, Fragment2 (2′,3′-cyclic phosphate) C_96_H_118_N_40_O_68_P_10_ [M]^−2^ calcd 3230, found 3229. **RNA13** (-AUAA-) Fragment1 C_47_H_61_N_19_O_32_P_4_ [M]^−2^ calcd 1528, found 1527, Fragment2 (2′,3′-cyclic phosphate) C_96_H_117_N_39_O_69_P_10_ [M]^−2^ calcd 3231, found 3230. **RNA24** (-AUA-) Fragment1 C_47_H_61_N_19_O_32_P_4_ [M]^−2^ calcd 1528, found 1527, Fragment2 (2′,3′-cyclic phosphate) C_86_H_105_N_34_O_63_P_9_ [M]^−2^ calcd 2902, found 2901. **RNA32** (-GUA-) Fragment1 C_47_H_61_N_19_O_32_P_4_ [M]^−2^ calcd 1528, found 1527, Fragment2 (2′,3′-cyclic phosphate) C_86_H_105_N_34_O_64_P_9_ [M]^−2^ calcd 2918, found 2917.

## 4. Conclusions

We, for the first time, report on how the sequence of an RNA bulge formed upon hybridization to a Zn^2+^-dependent PNAzyme affects the rate/efficiency of cleavage as well as site selectivity of the RNA cleavage. For 4-nt bulges formed and cleaved, this can lead to both substantial rate enhancement (from a starting point of 11–12 h to 7–8 h half-life) and increased site selectivity (from 50–60% to over 70%). Three-nt bulges in previous studies were cleaved less efficiently by Zn^2+^-based PNAzymes than 4-nt bulges [26]. However, here we show that 3-nt bulges can be cleaved at a similar rate and the enhancement obtained by bulge variation was more pronounced (from 21–24 h half-life down to 7–8 h half-life). In addition, the cleavage of several 3-nt bulges were even more site-selective (up to over 80% site selectivity) than for the most selective cleavages of 4-nt bulges. The studied Zn^2+^ PNAzyme is still less efficient than the most active Cu^2+^-dependent PNAzymes, but the present study demonstrates that for Zn^2+^-dependent PNAzymes carrying novel chelates, not only the “cleaving unit” is important, but it is also essential to investigate different potential RNA targets in order to find optimal PNAzyme action. This should be even more important as PNAzymes are developed for use in cell assays and eventually in vivo. 

## Figures and Tables

**Table 1:** Extent of cleavage of the different 4-nucleotide bulge forming RNA sequences **1**–**22** in the presence of **PNAzyme1** ^a^.

RNA	RNA Sequence	% of Cleavage	
		3 h	24 h
	nt nr: 8, 7, 6, 5		
**1**	5′-*AGAGUUC*-AAAA-*GCCC*-3′	18	75
**2**	-AAAG-	12	58
**3**	-AAAC-	7	41
**4**	-AAAU-	9	42
**5**	-AAGA-	14	70
**6**	-AACA-	4	24
**7**	-AAUA-	2	13
**8**	-AAGG-	10	53
**9**	-AACC-	2	15
**10**	-AAUU-	5	32
**11**	-AGAA-	19	78
**12**	-ACAA-	26	84
**13**	-AUAA-	32	91
**14**	-GAAA-	19	78
**15**	-CAAA-	29	89
**16**	-UAAA-	25	85
**17**	-UUAA-	28	88
**18**	-UUUA-	13	58
**19**	-UAUA-	10	57
**20**	-AUGA-	25	87
**21**	-ACGA-	20	82
**22**	-CUGA-	23	80

^a^ For sequences **2**–**22**, only the varied RNA-bulge is indicated. Reactions were carried out with a 1:1 ratio of **PNAzyme1** and RNA target, (4 μM each) at 10 mM HEPES buffer pH 7.4, 0.1 M NaCl at 37 °C and in the presence of 100 μM Zn^2+^. The stability constant for the zinc-neocuproine complex is only about 10^4^, which means that an excess of zinc ions is necessary to keep the chelate nearly saturated [29]. Values are reported as the means of duplicate or triplicate measurements. In these conditions, single stranded RNA1 is cleaved at a rate of 2 × 10^−6^ s^−1^, and in the presence of one equivalent of non-conjugated PNA1 (without neocuproine moiety), the rate of cleavage is less than 1 × 10^−6^ s^−1^ [26]. In general, the extent of cleavage at the two different time points is consistent with first order reactions.

**Table 2:** Extent of cleavage of the different 3-nucleotide bulges forming RNA sequences **23**–**37** in the presence of **PNAzyme1** ^a^.

RNA	RNA Sequence	% of Cleavage	
		3 h	24 h
	nt nr: 7, 6, 5		
**23**	5′-*AGAGUUC*-AAA-*GCCC*-3′	11	56
**24**	-AUA-	18	76
**25**	-AGA-	13	66
**26**	-ACA-	7	45
**27**	-AAU-	6	36
**28**	-UAA-	23	86
**29**	-AUG-	9	47
**30**	-AUC-	5	30
**31**	-AUU-	6	37
**32**	-GUA-	29	91
**33**	-CUA-	24	84
**34**	-UUA-	30	91
**35**	-GUG-	12	61
**36**	-CUC-	7	41
**37**	-ACG-	5	29

^a^ For sequences **24**–**37**, only the varied RNA-bulge is indicated. Reactions were carried out with a 1:1 ratio of **PNAzyme1** and RNA target, (4 μM each) at 10 mM HEPES buffer pH 7.4, 0.1 M NaCl at 37 °C and in the presence of 100 μM Zn^2+^. The stability constant for the zinc-neocuproine complex is only about 10^4^, which means that an excess of zinc ions is necessary to keep the chelate nearly saturated [29]. Values are reported as the means of duplicate or triplicate measurements. In general, the extent of cleavage at the two different time points is consistent with first order reactions.

## Supplementary Materials

The following are available online, Figure S1: Ion Exchange HPLC chromatograms of PNAzyme-assisted cleavage of **RNA1**–**22**, Figure S2: Ion Exchange HPLC chromatograms of PNAzyme-assisted cleavage of **RNA23**–**37**.

## References

[1] Lundin K.E., Gissberg O., Smith C.I.E. (2015). Oligonucleotide therapies: The past and the present. Hum. Gene Ther..

[2] Stein C.A., Castanotto D. (2017). FDA-approved oligonucleotide therapies in 2017. Mol. Ther..

[3] Sridharan K., Gogtay N.J. (2016). Therapeutic nucleic acids. Current clinical trials. Br. J. Clin. Pharmacol..

[4] Juliano R.L. (2016). The delivery of therapeutic oligonucleotides. Nucleic Acids Res..

[5] Kole R., Krainer A.R., Altman S. (2012). RNA therapeutics: Beyond RNA interference and antisense oligonucleotides. Nat. Rev. Drug Discov..

[6] Morrow J.R., Iranzo O. (2004). Synthetic metallonucleases for RNA cleavage. Curr. Opin. Chem. Biol..

[7] Ghidini A., Murtola M., Strömberg R., Stultz E. (2015). Oligonucleotide based artificial ribonucleases. DNA in Supramolecular Chemistry and Nanotechnology.

[8] Niittymäki T., Lönnberg H. (2006). Artificial ribonucleases. Org. Biomol. Chem..

[9] Laine M., Lönnberg T., Helkearo M., Lönnberg H. (2016). Cleavage of short oligoribonucleotides by a Zn^2+^ binding multi-nucleating azacrown conjugate. Inorg. Chim. Acta.

[10] Williams A., Staroseletz Y., Zenkova M.A., Jeannin L., Aojula H., Bichenkova E.V. (2015). Peptidyl-oligonucleotide conjugates demonstrate efficient cleavage of RNA in a sequence-specific manner. Bioconjugate Chem..

[11] Gnaccarini C., Peter S., Scheffer U., Vonhoff S., Klussmann S., Göbel M.W. (2006). Site-specific cleavage of RNA by a metal-free artificial nuclease attached to antisense oligonucleotides. J. Am. Chem. Soc..

[12] Hall J., Huesken D., Häner R. (1996). Towards artificial ribonucleases: The sequence-specific cleavage of RNA in a duplex. Nucleic Acids Res..

[13] Mikkola S., Kaukinen U., Lönnberg H. (2001). The effect of secondary structure on cleavage of the phosphodiester bonds of RNA. Cell Biochem. Biophys..

[14] Portmann S., Grimm S., Workman C., Usman N., Egli M. (1996). Crystal structures of an A-form duplex with single-adenosine bulges and a conformational basis for site-specific RNA self-cleavage. Chem. Biol..

[15] Hüsken D., Goodall G., Blommers M.J.J., Jahnke W., Hall J., Häner R., Moser H.E. (1996). Creating RNA bulges: Cleavage of RNA in RNA/DNA duplexes by metal ion catalysis. Biochemistry.

[16] Kaukinen U., Bielecki L., Mikkola S., Adamiak R.W., Lönnberg H. (2001). The cleavage of phosphodiester bonds within small RNA bulges in the presence and absence of metal ion catalysts. J. Chem. Soc. Perkin Trans. 2.

[17] Magda D., Wright M., Crofts S., Lin A., Sessler J.L. (1997). Metal complex conjugates of antisense DNA which display ribozyme-like activity. J. Am. Chem. Soc..

[18] Häner R., Hall J., Pfuezer A., Huesken D. (1998). Development of artificial ribonucleases. Pure Appl. Chem..

[19] Trawick B.N., Osiek T.A., Bashkin J.K. (2001). Enhancing sequence-specific cleavage of RNA within a duplex region: Incorporation of 1,3-propanediol linkers into oligonucleotide conjugates of serinol-terpyridine. Bioconjugate Chem..

[20] Åström H., Williams N.H., Strömberg R. (2003). Oligonucleotide based artificial nuclease (OBAN) systems. Bulge size dependence and positioning of catalytic group in cleavage of RNA-bulges. Org. Biomol. Chem..

[21] Åström H., Strömberg R. (2004). Synthesis of new OBAN′s and further studies on positioning of the catalytic group. Org. Biomol. Chem..

[22] Murtola M., Strömberg R. (2009). 2′-*O*-methyloligoribonucleotide based artificial nucleases (2′-O-MeOBANs) cleaving a model of the leukemia related M-BCR/ABL m-RNA. ARKIVOC.

[23] Danneberg F., Ghidini A., Dogandzhiyski P., Kalden E., Strömberg R., Göbel M.W. (2015). Sequence-specific RNA cleavage by PNA conjugates of the metal-free artificial ribonuclease tris(2-aminobenzimidazole). Beilstein J. Org. Chem..

[24] Sandbrink J., Murtola M., Strömberg R. (2007). Solid support post-conjugation of amino acids and a phenanthroline derivative to a central position in peptide nucleic acids. Nucleosides Nucleotides Nucleic Acids.

[25] Murtola M., Ossipov D., Sandbrink J., Strömberg R. (2007). RNA Cleavage by 2,9-diamino-1,10-phenanthroline PNA conjugates. Nucleosides Nucleotides Nucleic Acids.

[26] Murtola M., Strömberg R. (2008). PNA based artificial nucleases displaying catalysis with turnover in the cleavage of a leukemia related RNA model. Org. Biomol. Chem..

[27] Murtola M., Wenska M., Strömberg R. (2010). PNAzymes that are artificial RNA restriction enzymes. J. Am. Chem. Soc..

[28] Ghidini A., Bergquist H., Murtola M., Punga T., Zain R., Strömberg R. (2016). Clamping of RNA with PNA enables targeting of microRNA. Org. Biomol. Chem..

[29] Irving H., Mellor D.H. (1962). Stability of metal complexes of 1,10-phenanthroline and its analogs. I. 1,10-Phenanthroline and 2,2′-bipyridine. J. Chem. Soc..

[30] Åström H., Strömberg R. (2001). A method for synthesis of an artificial ribonuclease. Nucleosides Nucleotides Nucleic Acids.

[31] Puglisi J.D., Tinoco I. (1989). Absorbance melting curves of RNA. Methods Enzymol..

