# Supplementary material for: Zinc Ion-Dependent Peptide Nucleic Acid-Based Artificial Enzyme that Cleaves RNA—Bulge Size and Sequence Dependence
Source: Molecules. 2017 Oct 29;22(11):1856. doi: 10.3390/molecules22111856 (PMC6150328; doi:10.3390/molecules22111856)

Supplementary

Figure S1. IE-HPLC analysis of 4-nucleotide bulge RNA cleavage reactions in the presence of **PNAzyme1** and RNA (1:1, PNAzyme:RNA). Reactions were carried out using 100 μM effective concentration of Zn^{2+} and were analyzed at the 24 h time point (pH 7.4, 37 °C).

RNA1, (4-nucleotide bulge: 5´-AAAA-3´)

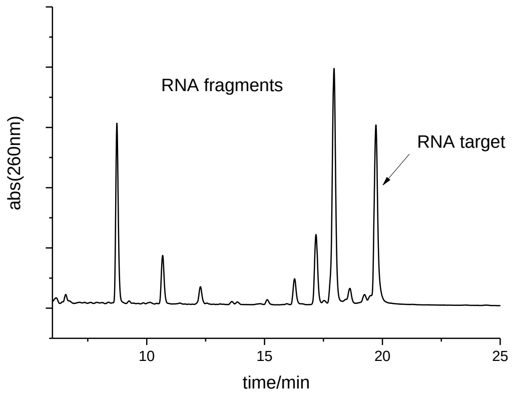

RNA2, (4-nucleotide bulge: 5´-AAAG-3´)

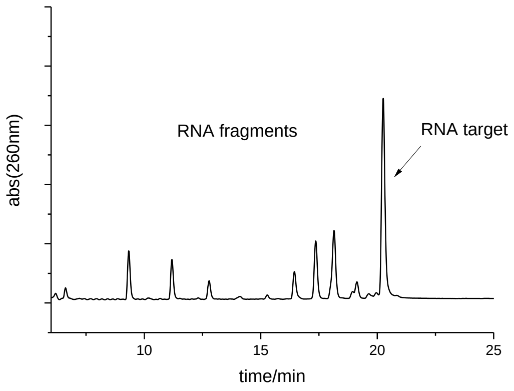

RNA3, (4-nucleotide bulge: 5´-AAAC-3´)

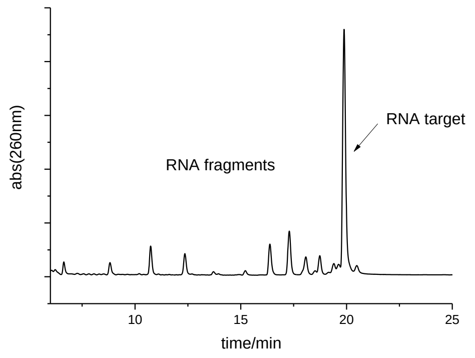

RNA4, (4-nucleotide bulge: 5´-AAAU-3´)

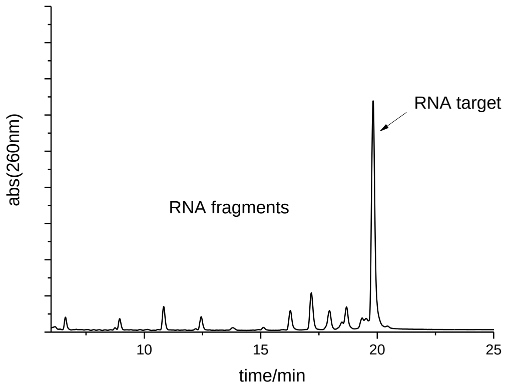

RNA5, (4-nucleotide bulge: 5´-AAGA-3´)

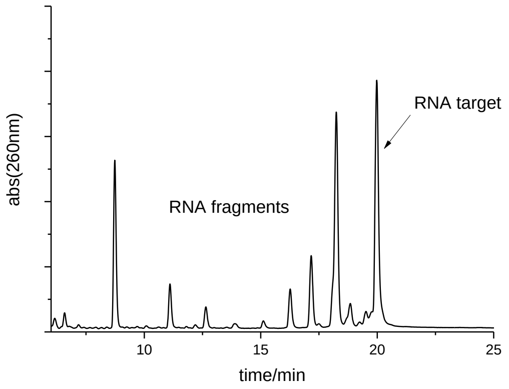

RNA6, (4-nucleotide bulge: 5´-AACA-3´)

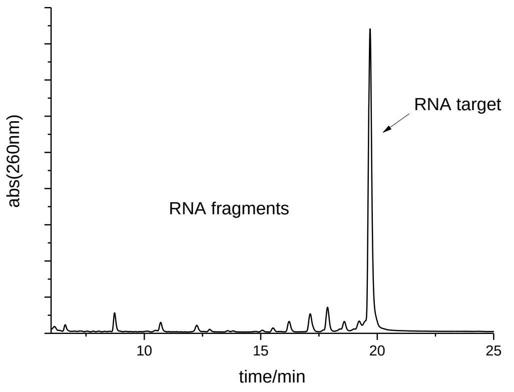

RNA7, (4-nucleotide bulge: 5´-AAUA-3´)

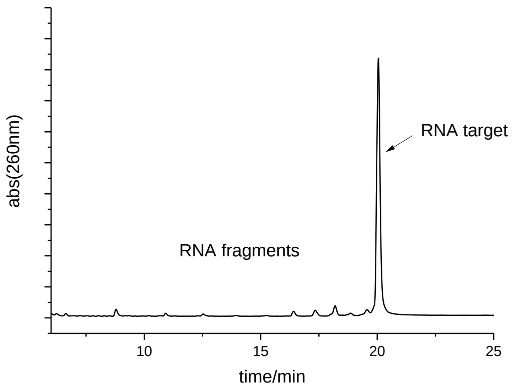

RNA8, (4-nucleotide bulge: 5´-AAGG-3´)

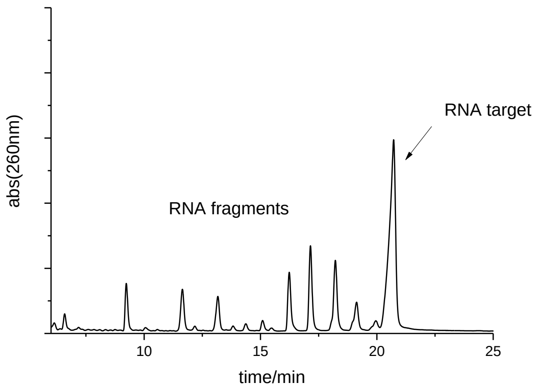

RNA9, (4-nucleotide bulge: 5´-AACC-3´)

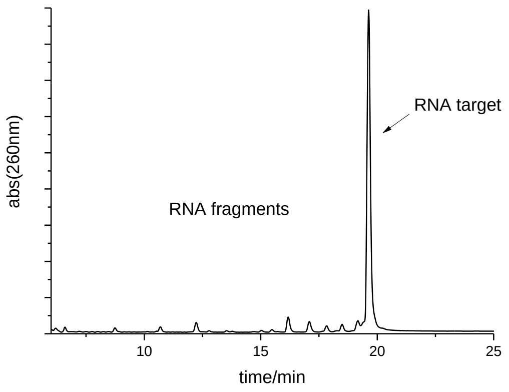

RNA10, (4-nucleotide bulge: 5´-AAUU-3´)

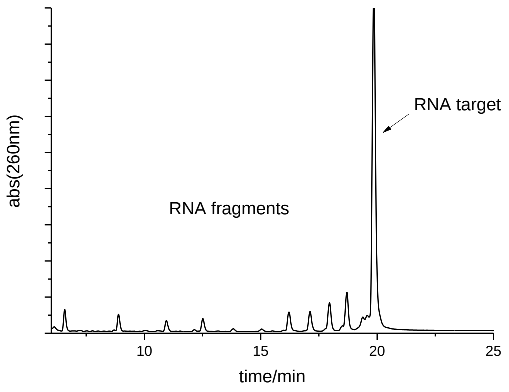

RNA11, (4-nucleotide bulge: 5´-AGAA-3´)

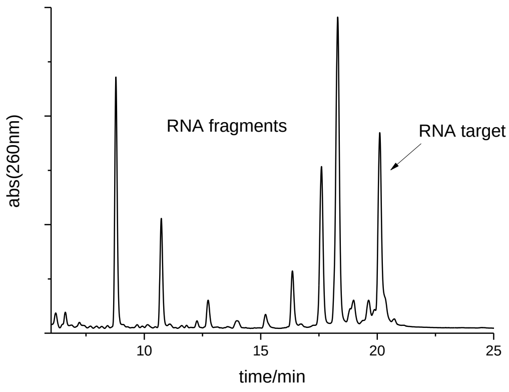

RNA12, (4-nucleotide bulge: 5´-ACAA-3´)

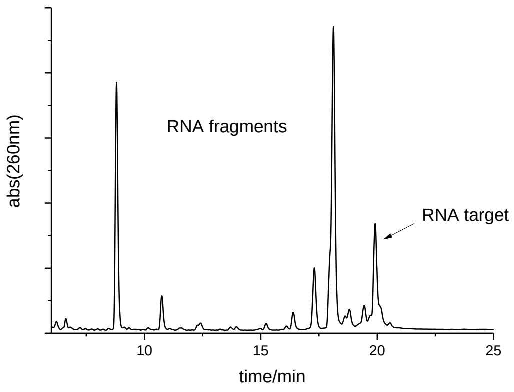

RNA13, (4-nucleotide bulge: 5´-AUAA-3´)

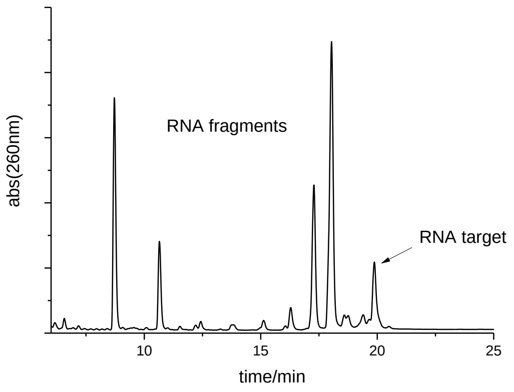

RNA14, (4-nucleotide bulge: 5´-GAAA-3´)

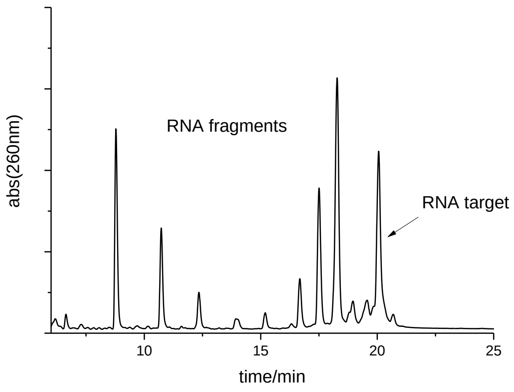

RNA15, (4-nucleotide bulge: 5´-CAAA-3´)

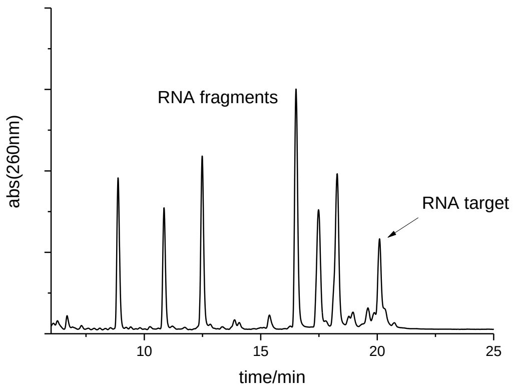

RNA16, (4-nucleotide bulge: 5´-UAAA-3´)

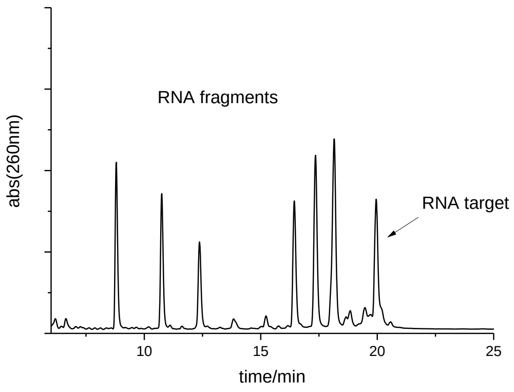

RNA17, (4-nucleotide bulge: 5´-UUAA-3´)

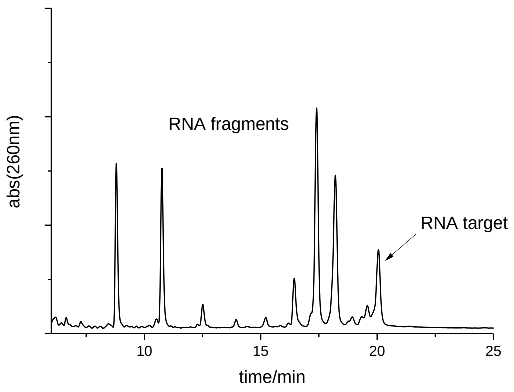

RNA18, (4-nucleotide bulge: 5´-UUUA-3´)

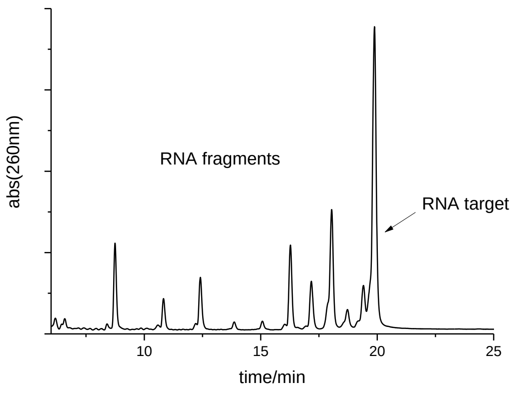

RNA19, (4-nucleotide bulge: 5´-UAUA-3´)

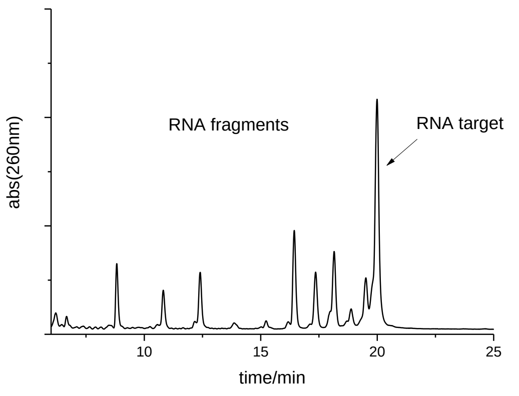

RNA20, (4-nucleotide bulge: 5´-AUGA-3´)

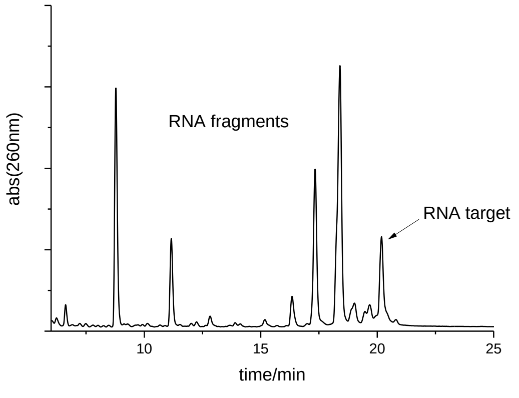

RNA21, (4-nucleotide bulge: 5′-ACGA-3′)

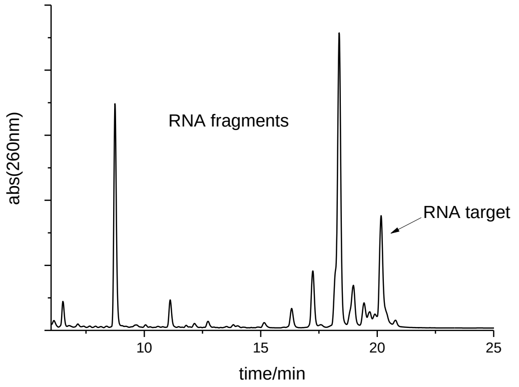

RNA22, (4-nucleotide bulge: 5′-CUGA-3′)

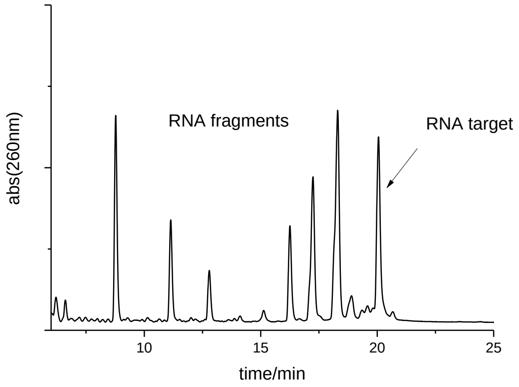

Figure S2. IE-HPLC analysis of 3-nucleotide bulge RNA cleavage reactions in the presence of **PNAzyme1** and RNA (1:1, PNAzyme:RNA). Reactions were carried out using 100 μM effective concentration of Zn^{2+} and were analyzed at the 24 h time point (pH 7.4, 37 °C).

RNA23, (3-nucleotide bulge: 5´-AAA-3´)

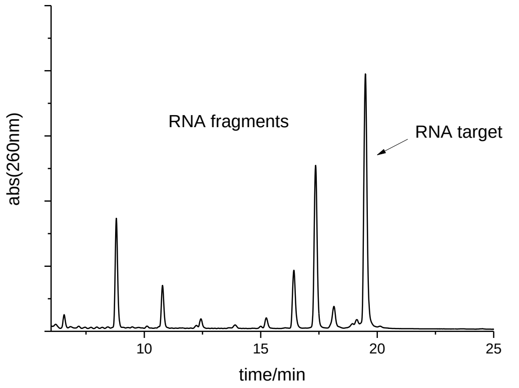

RNA24, (3-nucleotide bulge: 5´-AUA-3´)

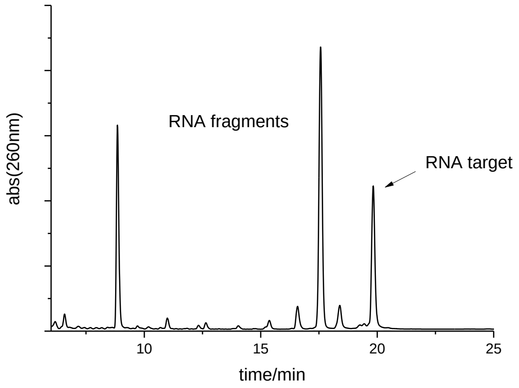

RNA25, (3-nucleotide bulge: 5´-AGA-3´)

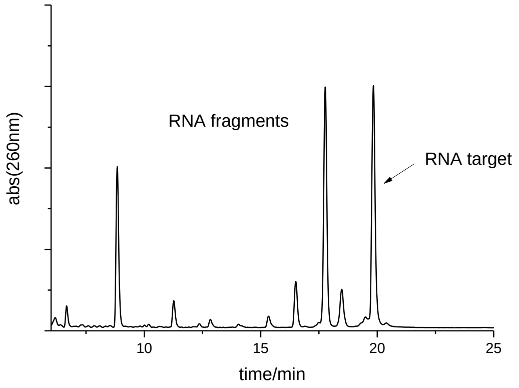

RNA26, (3-nucleotide bulge: 5´-ACA-3´)

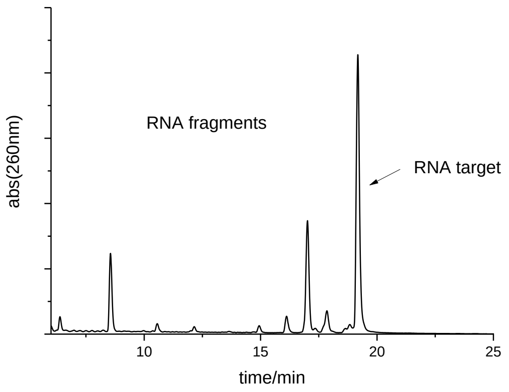

RNA27, (3-nucleotide bulge: 5´-AAU-3´)

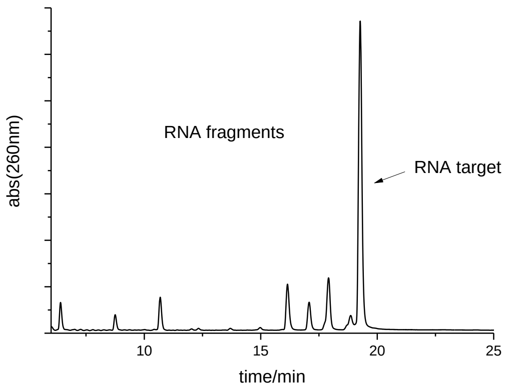

RNA28, (3-nucleotide bulge: 5´-UAA-3´)

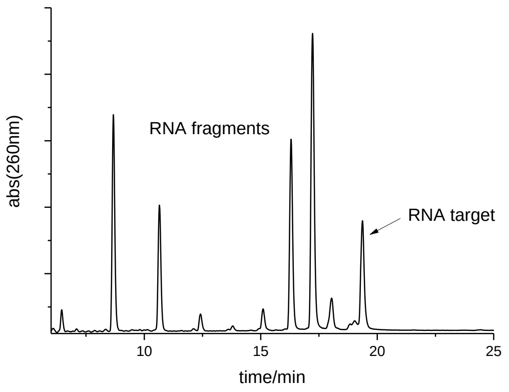

RNA29, (3-nucleotide bulge: 5´-AUG-3´)

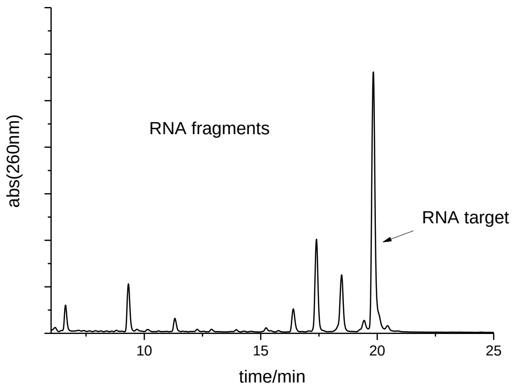

RNA30, (3-nucleotide bulge: 5´-AUC-3´)

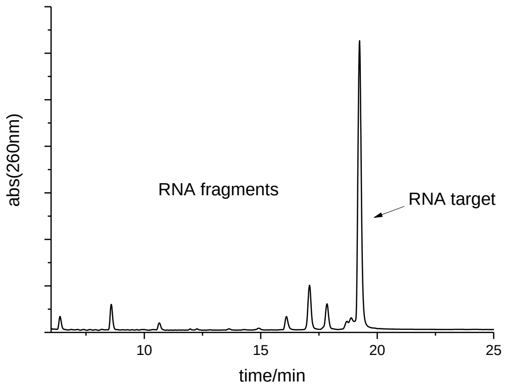

RNA31, (3-nucleotide bulge: 5´-AUU-3´)

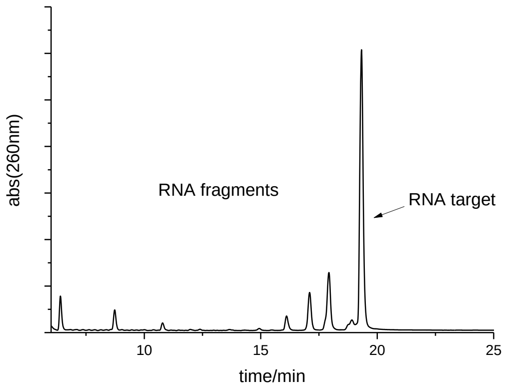

RNA32, (3-nucleotide bulge: 5´-GUA-3´)

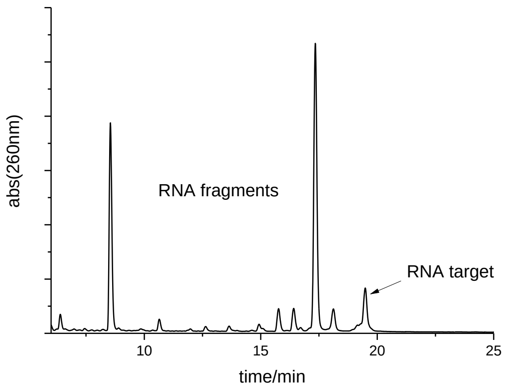

RNA33, (3-nucleotide bulge: 5´-CUA-3´)

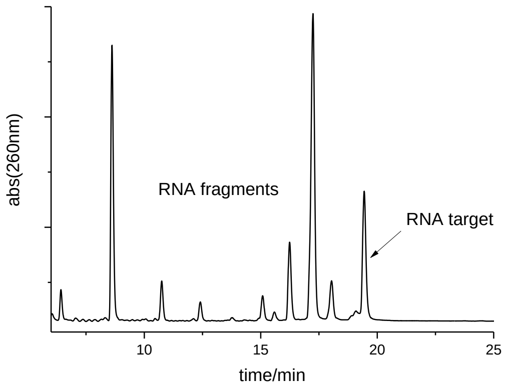

RNA34, (3-nucleotide bulge: 5´-UUA-3´)

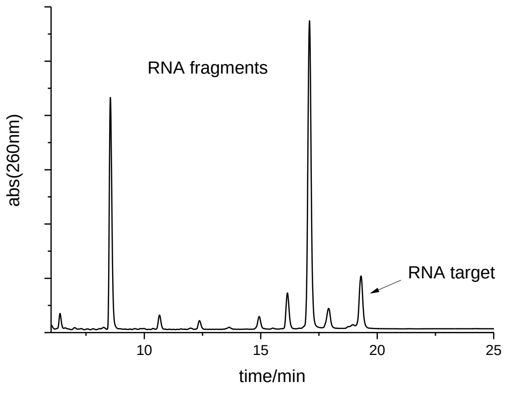

RNA35, (3-nucleotide bulge: 5´-GUG-3´)

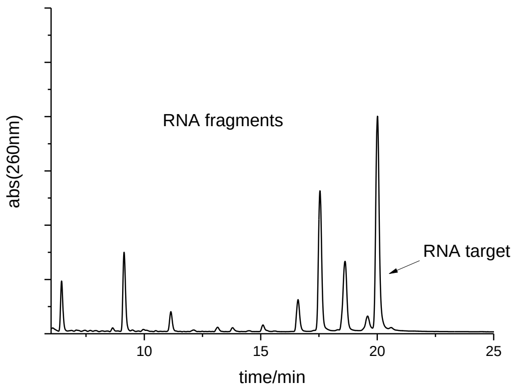

RNA36, (3-nucleotide bulge: 5´-CUC-3´)

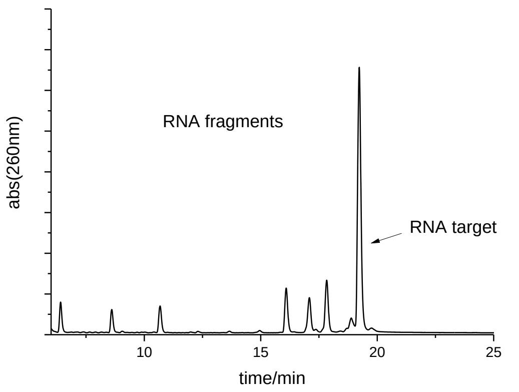

RNA37, (3-nucleotide bulge: 5´-ACG-3´)

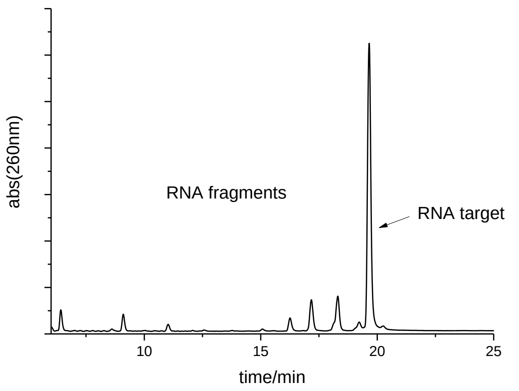

Supplement: Supplementary file 1 [file molecules-22-01856-s001.pdf]
